# Evaluating Age-Adjusted D-dimer and Clinical Decision-Making in Suspected Pulmonary Embolism: A Retrospective Clinical Audit and Practitioner Survey

**DOI:** 10.7759/cureus.117406

**Published:** 2026-10-03

**Authors:** Jinsha Khan Basheer, Akhil Jerry, Jhanvi Sawlani, Maher Mahbub, Abdul M Shafi

**Affiliations:** 1 Acute Medicine, Queen Elizabeth Hospital, Birmingham, GBR; 2 General Internal Medicine, Queen Elizabeth Hospital, Birmingham, GBR; 3 Diabetes and Endocrinology, Queen Elizabeth Hospital, Birmingham, GBR; 4 Internal Medicine, Queen Elizabeth Hospital, Birmingham, GBR

**Keywords:** age-adjusted d-dimer, ct pulmonary angiogram (ctpa), d-dimer cut off value, lack of health care provider knowledge, pulmonary embolism (pe)

## Abstract

Background

Pulmonary embolism (PE) is a common and potentially life-threatening condition encountered in acute medical practice. Computed tomography pulmonary angiography (CTPA) remains the standard imaging modality for diagnosis, but increasing utilisation raises concerns regarding radiation exposure, contrast-related complications, and healthcare resource use. This study evaluated diagnostic pathways for suspected PE through a retrospective clinical audit and practitioner surveys conducted before and after an educational intervention.

Methods

This study comprised a retrospective clinical audit of 1499 consecutive patients who underwent CTPA for suspected PE, an initial cross-sectional survey of 48 healthcare professionals, and a repeat cross-sectional survey of 33 healthcare professionals conducted after an educational intervention. Collected variables included demographics, Wells score, National Early Warning Score 2 (NEWS2), oxygen saturation, heart rate, D-dimer results, CTPA findings, alternative diagnoses, and in-hospital mortality. The practitioner surveys assessed knowledge of D-dimer testing, use of age-adjusted thresholds, and factors influencing clinical decision-making. Of 1499 CTPA episodes, 1150 had an interpretable D-dimer result. Eight episodes without a definitive CTPA reference-standard outcome were excluded, leaving 1142 episodes for the diagnostic-performance analysis.

Results

PE was confirmed in 206 of 1499 patients, giving an overall prevalence of 13.7%. Among 1142 episodes included in the diagnostic-performance analysis, the standard threshold demonstrated a sensitivity of 87.8% (95% CI 82.3%-91.7%) and specificity of 14.8% (95% CI 12.7%-17.2%). The age-adjusted threshold demonstrated a sensitivity of 86.2% (95% CI 80.5%-90.4%) and specificity of 23.4% (95% CI 20.8%-26.2%). False-positive classifications decreased from 813 to 731, an absolute reduction of 82 cases and a relative reduction of 10.1%, while false-negative classifications increased from 23 to 26.

The initial practitioner survey identified important knowledge gaps. Age-adjusted D-dimer was used regularly by 21 (44%) of respondents and occasionally by 13 (27%), while six (12%) were unfamiliar with the concept. Following the educational intervention, regular use increased to 18 (54.5%), occasional use was reported by eight (24.2%), and unfamiliarity decreased to approximately one (3%). Awareness of the local D-dimer assay also improved.

Clinical decision-making was influenced by fear of missing PE in 32/48 (66.7%) of respondents, while 24/48 (50.0%) reported that clinical impression could outweigh formal scoring systems. In the initial survey, 29/48 respondents (60.4%) were unsure which D-dimer assay was used locally.

Conclusions

Retrospective application of the age-adjusted threshold in this CTPA-selected cohort increased specificity and reduced false-positive classifications, with a small reduction in sensitivity and three additional false-negative classifications. Because the analysis was not restricted to patients with PE-unlikely or non-high pre-test probability and did not assess actual imaging utilisation or follow-up safety, these findings do not establish the safety of age-adjusted D-dimer as a PE rule-out strategy. The repeat practitioner survey demonstrated higher assay knowledge and self-reported use; however, causality cannot be inferred from the unpaired survey design.

## Introduction

Pulmonary embolism (PE) is a common cause of morbidity and mortality and remains an important diagnostic challenge in acute clinical settings. Despite being one of the most common presentations encountered during the acute medical take, its varied and sometimes nonspecific clinical manifestations can make timely diagnosis difficult.

In the United Kingdom, the estimated annual incidence of diagnosed PE is approximately 7-8 per 10,000 individuals. National data have demonstrated a doubling of hospital admissions for PE between 1997 and 2015. This increase is likely attributable, at least partly, to greater utilisation of computed tomography pulmonary angiography (CTPA) and improved detection rather than a true increase in disease burden [1,2]. Internationally, the reported incidence of PE ranges from approximately 50 to 100 cases per 100,000 person-years, with age and comorbidity being important determinants [3,4]. Mortality data from the World Health Organization indicate that PE accounts for approximately 0.46% of all recorded deaths globally, although underdiagnosis remains an important concern [5].

These findings highlight the continuing clinical significance of PE as a frequent and potentially preventable cause of morbidity and mortality. Over several decades, efforts to standardise the diagnostic approach to suspected PE have led to structured pathways incorporating assessments of clinical probability, such as the Wells score, D-dimer testing, and CTPA [6,7]. However, because PE can present with a wide range of symptoms and clinical findings, CTPA utilisation continues to increase in clinical practice, raising concerns regarding radiation exposure, contrast-associated complications, and pressure on healthcare resources [8].

D-dimer testing has high sensitivity but limited specificity, particularly in older adults, in whom baseline fibrin degradation product levels may be elevated. To improve diagnostic specificity while maintaining sensitivity, age-adjusted D-dimer thresholds have been validated in large multicentre studies. Commonly used thresholds include age × 10 ng/mL for patients older than 50 years when fibrinogen equivalent units (FEU) are used, or age × 5 ng/mL when D-dimer units (DDU) are used [9]. National guidance, including the National Institute for Health and Care Excellence guideline NG158, and relevant professional recommendations support the use of pre-test probability assessment and D-dimer testing before proceeding to diagnostic imaging where appropriate [6,7].

Despite established evidence and guideline recommendations, implementation of age-adjusted D-dimer thresholds in routine clinical practice may remain inconsistent. While previous studies have extensively evaluated the diagnostic performance of age-adjusted D-dimer, comparatively less attention has been given to clinicians' knowledge, awareness, and decision-making processes influencing its use in real-world settings.

The primary objective was to compare the diagnostic classification performance of standard and age-adjusted D-dimer thresholds among patients undergoing CTPA for suspected PE. The secondary objectives were to assess clinicians' knowledge and self-reported use of age-adjusted D-dimer in two unpaired cross-sectional surveys conducted before and after an educational intervention.

## Materials and methods

This study comprised a retrospective clinical audit and two cross-sectional practitioner surveys conducted at Queen Elizabeth Hospital Birmingham. The clinical audit evaluated consecutive patients undergoing computed tomography pulmonary angiography (CTPA) for suspected pulmonary embolism (PE). The practitioner component comprised an initial cross-sectional questionnaire assessing clinicians’ knowledge and self-reported use of D-dimer testing and age-adjusted D-dimer thresholds, followed by a repeat cross-sectional survey conducted after an educational intervention. The two survey samples were anonymous and unpaired. An overview of the retrospective clinical audit, practitioner surveys and educational intervention is shown in Figure 1.

**Figure 1:**
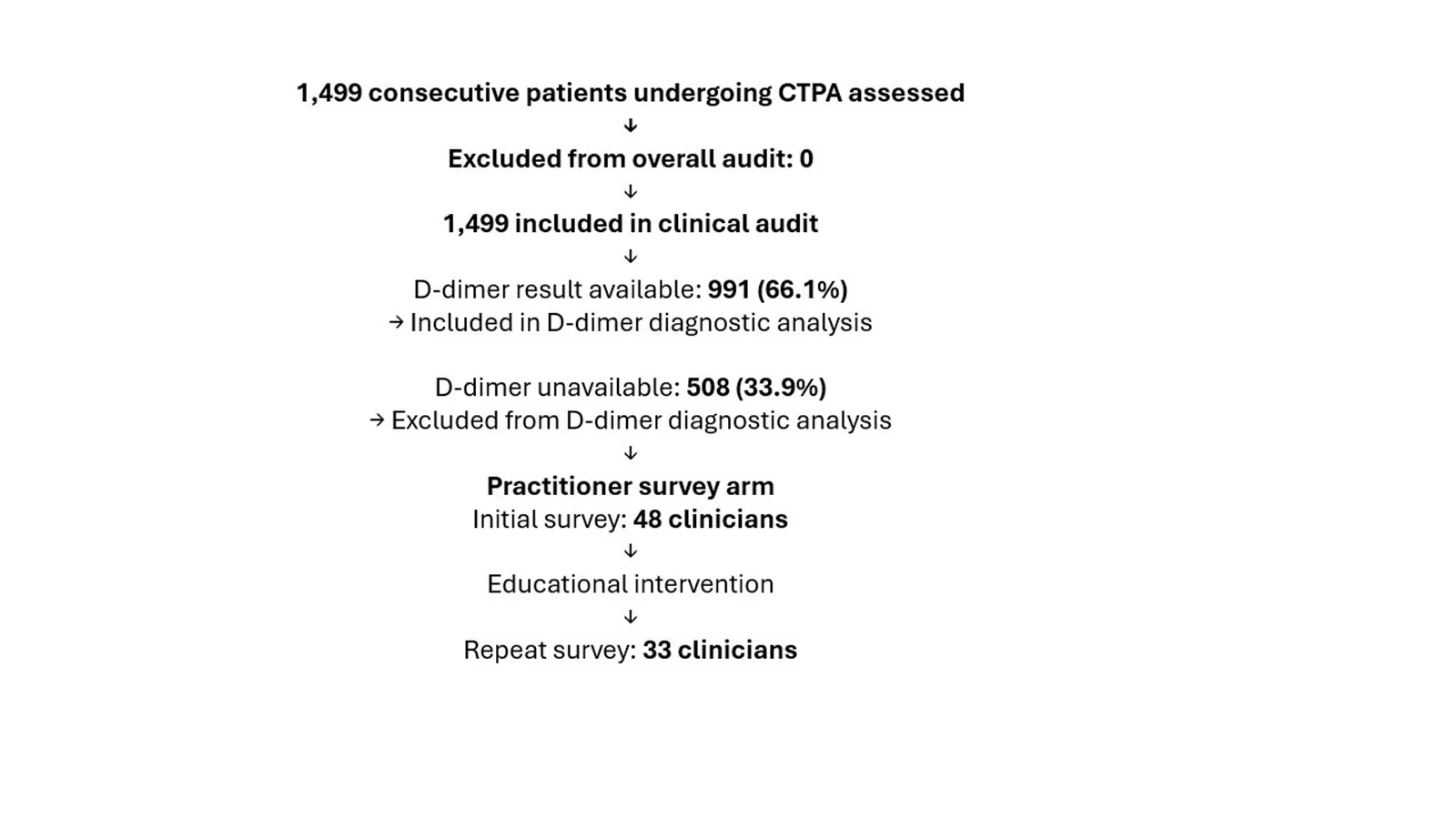
Study design and practitioner survey pathway Flow diagram illustrating the study design. The study comprised a retrospective clinical audit of 1499 patients undergoing CTPA for suspected PE, an initial cross-sectional clinician survey (n=48), a targeted educational intervention, and a repeat cross-sectional clinician survey (n=33). CTPA - computed tomography pulmonary angiography; PE - pulmonary embolism; DDU - D-dimer units Image Credit: Jinsha Khan Basheer. Figure created using PowerPoint (Microsoft, Redmond, Washington).

Clinical Audit

Setting

The clinical audit was conducted at Queen Elizabeth Hospital Birmingham, a tertiary referral centre utilising D-dimer unit (DDU) assays with a standard diagnostic cut-off of 250 ng/mL.

Population

All consecutive patients who underwent computed tomography pulmonary angiography (CTPA) for suspected pulmonary embolism (PE) between January and March 2024 were included in the audit. A total of 1499 patients were included during the three-month study period.

Eligibility Criteria

Diagnostic-performance analyses included episodes with an interpretable D-dimer result, sufficient age data to determine the applicable threshold, and a definitive CTPA reference-standard outcome. D-dimer results were considered interpretable when reported either as a numerical value or as a censored value below the assay reporting limit, such as <150, <250, or <75 µg/L DDU. No point value was imputed for censored results; they were classified as negative when the reported value was below the applicable decision threshold. Eight episodes without a definitive reference-standard outcome were excluded. No imputation was performed for other missing data.

Data Collection

Clinical and demographic data were collected retrospectively from the hospital informatics department and electronic patient records. Extracted variables included patient demographics, Wells score [10], National Early Warning Score 2 (NEWS2) [11], oxygen saturation, heart rate, D-dimer result where available, CTPA outcome, alternative diagnoses, and in-hospital mortality. Where information was not available in the informatics dataset, relevant data were manually extracted from the electronic patient record system.

Practitioner survey

A cross-sectional questionnaire was distributed to healthcare professionals involved in the assessment and management of patients with suspected PE across multiple specialities. 

The survey instrument was piloted with five clinicians before wider distribution. Forty-eight healthcare professionals participated in the initial survey, comprising junior doctors 17 (35.4%), senior house officers 19 (39.6%), registrars nine (18.8%), advanced clinical practitioners two (4.2%), and consultants one (2.1%). The questionnaire explored D-dimer utilisation, awareness of age-adjusted D-dimer thresholds, factors influencing clinical decision-making, challenges associated with CTPA requesting, and educational needs. Following the initial survey, a targeted educational awareness intervention was undertaken focusing on the local D-dimer threshold, interpretation of age-adjusted D-dimer values, awareness of the locally used DDU assay, and evidence-based PE diagnostic pathways. For patients with more than one D-dimer result, the most recent D-dimer measurement obtained before CTPA was used. The retrospective D-dimer classification analysis was not consistently restricted to patients with PE-unlikely or non-high clinical probability because complete pre-test probability data were not available for all patients. The analysis should therefore not be interpreted as validation of a D-dimer rule-out pathway.

A repeat survey was subsequently conducted among 33 healthcare professionals to assess changes in knowledge and reported clinical practice following the intervention.

D-dimer analysis

D-dimer was measured using a latex agglutination assay and reported in µg/L D-dimer units (DDU). The standard institutional threshold was 250 µg/L DDU. A result of ≥250 µg/L DDU was classified as positive using the standard threshold. For the age-adjusted analysis, the threshold remained 250 µg/L DDU for patients aged ≤50 years and was calculated as age × 5 µg/L DDU for patients aged >50 years. For patients with more than one D-dimer measurement, the most recent result obtained before CTPA was used. CTPA classification was used as the reference standard.

Statistical analysis

Descriptive variables were summarised using counts and percentages or means with standard deviations, as appropriate. Diagnostic performance of the standard and age-adjusted D-dimer thresholds was assessed using 2 × 2 contingency tables. Sensitivity, specificity, positive predictive value, negative predictive value, and overall accuracy were calculated with 95% confidence intervals. Patients with missing values required for a specific analysis were excluded from that analysis, and denominators are reported explicitly. Equivocal CTPA examinations were excluded from diagnostic-performance calculations. Survey data were analysed descriptively as n/N (%). Because the initial and repeat surveys comprised independent, unpaired samples, differences were interpreted as group-level differences and not as paired individual change.

Patient and public involvement

Patients and members of the public were not involved in the design, conduct, or reporting of the clinical audit and practitioner survey. Findings were intended for dissemination to the relevant clinical teams through departmental meetings and incorporation into local quality improvement initiatives.

Ethical considerations

The project was registered as a clinical audit with Queen Elizabeth Hospital Birmingham (Audit Registration Number: CRAMS-23955). Formal research ethics approval was not required according to the institutional classification and governance arrangements applicable to the project. Participation in the practitioner surveys was voluntary and anonymous.

Data availability

Anonymised patient-level data are available from the corresponding author upon reasonable request, subject to institutional approval and applicable data-sharing agreements.

## Results

CTPA findings and baseline characteristics

Between January and March 2024, 1499 consecutive patients underwent computed tomography pulmonary angiography (CTPA) for suspected pulmonary embolism (PE). PE was confirmed in 206 patients (13.7%), excluded in 1288 patients (85.9%), and reported as equivocal in five patients (0.3%). The mean age of the cohort was 60.2 years. No significant difference in PE prevalence was observed between genders.

Wells Score

The mean Wells score was significantly higher in patients with confirmed PE than in those without PE (5.02 ± 1.96 vs 4.70 ± 1.63; p=0.004). However, the relatively small difference between groups suggests limited discriminatory value when the Wells score is considered in isolation.

Physiological Parameters

PE positivity varied according to physiological parameters. NEWS2 data were available for 1396/1499 patients (93.1%). PE was identified in 107/812 (13.2%) patients with a NEWS2 score of 0-4, 49/262 (18.7%) with a NEWS2 score of 5-6, 39/207 (18.8%) with a NEWS2 score of 7-9, and 11/115 (9.6%) with a NEWS2 score of ≥10. Oxygen saturation data were available for 1442/1499 patients (96.2%). PE was identified in 91/733 (12.4%) patients with oxygen saturation ≥95%, 93/514 (18.1%) with oxygen saturation of 90%-94%, and 18/195 (9.2%) with oxygen saturation <90%. Heart-rate data were available for 1440/1499 patients (96.1%). PE was detected in 97/781 (12.4%) patients with a heart rate ≤100 beats/min and 105/659 (15.9%) with a heart rate >100 beats/min. The distribution of PE positivity across NEWS2, oxygen saturation, and heart-rate categories is summarised in Table 1.

**Table 1:** PE positivity according to physiological parameters NEWS2 was available for 1396/1499 patients, oxygen saturation for 1442/1499, and heart rate for 1440/1499. Percentages represent PE-positive patients within each subgroup.

Variable	Subgroup	PE positive / N	PE positivity
NEWS2	0–4	107/812	13.20%
	5–6	49/262	18.70%
	7–9	39/207	18.80%
	≥10	11/115	9.60%
Oxygen saturation	≥95%	91/733	12.40%
	90–94%	93/514	18.10%
	<90%	18/195	9.20%
Heart rate	≤100 beats/min	97/781	12.40%
	>100 beats/min	105/659	15.90%

The highest observed PE positivity was in the NEWS2 7-9 category (18.8%), although a similar proportion was observed in the NEWS2 5-6 category (18.7%). Patients with a NEWS2 score of ≥10 had a lower observed PE positivity rate (9.6%) than those in the intermediate NEWS2 categories. These findings are descriptive and should not be interpreted as demonstrating an independent association between NEWS2 and PE.

Of the 1499 records, 1150 (76.7%) had an interpretable D-dimer result. These comprised 1093 numerical results and 57 censored results reported below the laboratory measurement threshold, such as <150, <250 or <75 µg/L DDU. D-dimer results were unavailable or uninterpretable in 349/1499 records (23.3%). Eight of the 1150 records did not have a definitive CTPA reference-standard outcome and were excluded, leaving 1142 records for the diagnostic-performance analysis. The availability of interpretable D-dimer results is shown in Figure 2.

**Figure 2:**
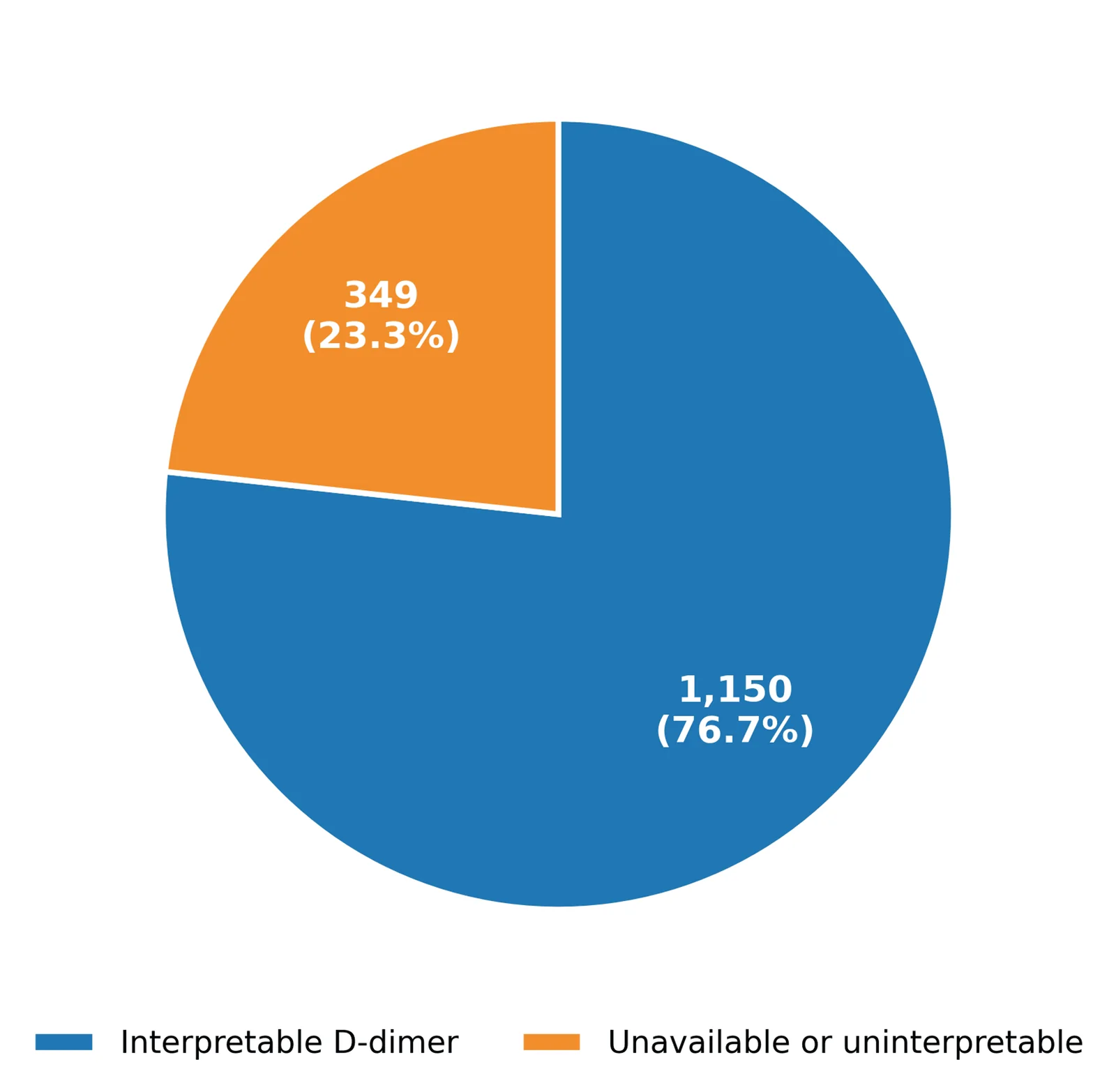
Availability of Interpretable D-dimer results Distribution of interpretable D-dimer results within the retrospective cohort. An interpretable D-dimer result was available in 1150/1499 records (76.7%), while the result was unavailable or uninterpretable in 349/1499 records (23.3%).

Among the 1142 records included in the diagnostic-performance analysis, the standard D-dimer threshold demonstrated a sensitivity of 87.8% (95% CI 82.3%-91.7%) and a specificity of 14.8% (95% CI 12.7%-17.2%). The age-adjusted threshold demonstrated a sensitivity of 86.2% (95% CI 80.5%-90.4%) and a specificity of 23.4% (95% CI 20.8%-26.2%). False-positive classifications decreased from 813 to 731, representing 82 fewer false-positive classifications and a relative reduction of 10.1%. The false-positive rate decreased from 85.2% to 76.6%, an absolute reduction of 8.6 percentage points. False-negative classifications increased from 23 to 26. The classification of standard and age-adjusted D-dimer results against the CTPA reference standard is presented in Tables 2 and 3.

**Table 2:** Classification of standard D-dimer results against CTPA outcome Classification using the standard D-dimer threshold of 250 µg/L DDU. The diagnostic-analysis cohort comprised 1142 records, including 188 PE-positive and 954 PE-negative records. CTPA classification was used as the reference standard. TP - true positive; FP - false positive; FN - false negative; TN - true negative; DDU - D-dimer units; CTPA - computed tomography pulmonary angiography

Standard D-dimer threshold	PE positive	PE negative
D-dimer positive	165 (TP)	813 (FP)
D-dimer negative	23 (FN)	141 (TN)

**Table 3:** Classification of age-adjusted D-dimer results against CTPA outcome Classification using a D-dimer threshold of 250 µg/L DDU for patients aged ≤50 years and age × 5 µg/L DDU for patients aged >50 years. The diagnostic-analysis cohort comprised 1,142 records, including 188 PE-positive and 954 PE-negative records. CTPA classification was used as the reference standard. TP - true positive; FP - false positive; FN - false negative; TN - true negative; DDU - D-dimer units; CTPA - computed tomography pulmonary angiography

Age-adjusted D-dimer threshold	PE positive	PE negative
D-dimer positive	162 (TP)	731 (FP)
D-dimer negative	26 (FN)	223 (TN)

Alternative Diagnoses

Among the 1288 PE-negative patients, an alternative diagnosis was identified in 1121 (87.0%), while no alternative pathology was identified in 167 (13.0%). The reported alternative diagnoses included systemic or non-pulmonary illness in 683/1288 cases (53.0%), pulmonary diagnoses in 322/1288 (25.0%), malignancy in 64/1288 (5.0%), and cardiac diagnoses in 39/1288 (3.0%). An additional 13/1288 cases (1.0%) were unclassified.

Mortality

In-hospital mortality occurred in 31/206 patients with confirmed PE (15.0%) and 153/1288 patients without PE (11.9%). The relative risk of in-hospital mortality among patients with PE was 1.26 (95% CI 0.89-1.79). The diagnostic performance of the standard and age-adjusted D-dimer thresholds, including sensitivity, specificity, accuracy, positive predictive value and negative predictive value, is summarised in Table 4. 

**Table 4:** Diagnostic performance of standard and age-adjusted D-dimer thresholds Diagnostic-performance estimates were calculated from 1142 records using CTPA classification as the reference standard. Confidence intervals were calculated using the Wilson score method. CI - confidence interval

Measure	Standard threshold	Age-adjusted threshold
Sensitivity	87.8% (95% CI 82.3%-91.7%)	86.2% (95% CI 80.5%-90.4%)
Specificity	14.8% (95% CI 12.7%-17.2%)	23.4% (95% CI 20.8%-26.2%)
Positive predictive value	16.9% (95% CI 14.7%-19.3%)	18.1% (95% CI 15.8%-20.8%)
Negative predictive value	86.0% (95% CI 79.8%-90.5%)	89.6% (95% CI 85.1%-92.8%)
Overall accuracy	26.8% (95% CI 24.3%-29.4%)	33.7% (95% CI 31.0%-36.5%)

Practitioner Survey

Initial Practitioner Survey

Forty-eight healthcare professionals participated in the initial practitioner survey, representing a range of clinical grades and specialities. Twenty-one of 48 respondents (43.8%) worked in Acute Medicine, and 13/48 (27.1%) worked in General Medicine.

Regular use of age-adjusted D-dimer thresholds was reported by 21/48 respondents (43.8%), occasional use by 13/48 (27.1%), non-use by 8/48 (16.7%), and unfamiliarity with the concept by 6/48 (12.5%). In addition, 29/48 respondents (60.4%) were unsure whether their institution used a D-dimer unit (DDU) or fibrinogen-equivalent unit (FEU) assay. Reported age thresholds for adjustment varied between 50 and 65 years, and errors were observed in scenario-based calculations. Detailed findings regarding clinicians’ knowledge of the local D-dimer threshold, awareness of the locally used assay, understanding of the age threshold for age-adjusted D-dimer testing, and responses to a case-based calculation question are presented in Figure 3.

**Figure 3:**
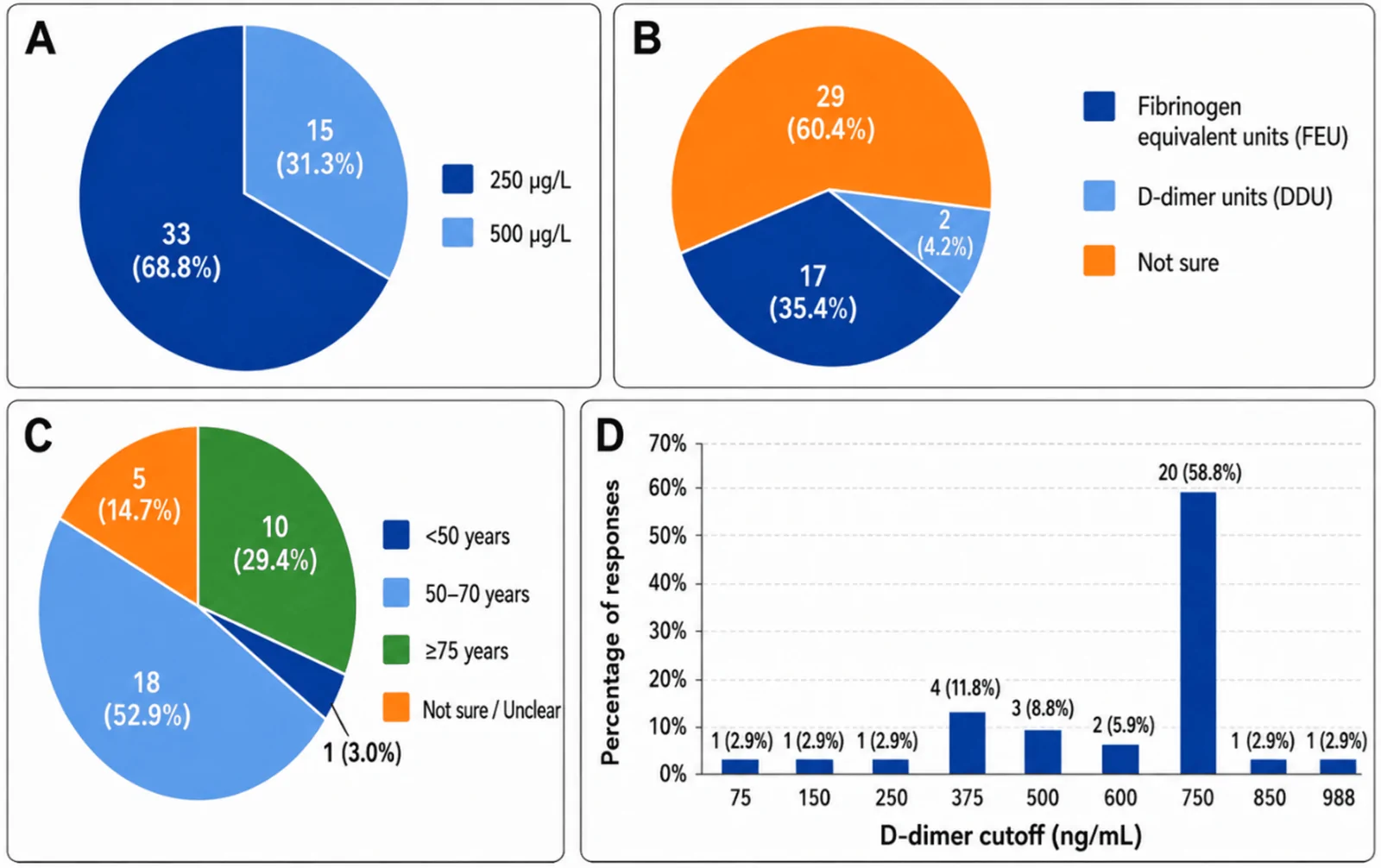
Initial clinician survey - knowledge of D-dimer A) Clinicians' knowledge of the local D-dimer cut-off used for suspected pulmonary embolism (PE); B) Clinicians' knowledge of the D-dimer assay used at the hospital; C) Clinicians' knowledge of the age threshold at which age-adjusted D-dimer calculations should be applied; D) Responses to a case-based question requiring calculation of the age-adjusted D-dimer cut-off for a 75-year-old woman with a low Wells score. FEU - fibrinogen equivalent units; DDU - D-dimer units; PE - pulmonary embolism Image credit: Jinsha Khan Basheer. Created using PowerPoint (Microsoft, Redmond, Washington).

Repeat Practitioner Survey Following the Educational Intervention

Following the educational intervention, 33 healthcare professionals participated in the repeat cross-sectional survey. Of these, 17/33 respondents (51.5%) worked in acute medicine, and 6/33 (18.2%) worked in general medicine.

Regular use of age-adjusted D-dimer thresholds was reported by 18/33 respondents (54.5%), occasional use by 8/33 (24.2%), non-use by 6/33 (18.2%), and unfamiliarity with the concept by 1/33 (3.0%). Twenty-one of 33 respondents (63.6%) correctly identified DDU as the assay used locally, and 23/33 (69.7%) correctly identified the local D-dimer threshold of 250 µg/L.

In the repeat survey, assay knowledge and self-reported use of age-adjusted D-dimer were higher than in the initial survey. However, because the surveys comprised independent, unpaired samples, these differences cannot be attributed causally to the educational intervention. A comparison of key findings from the initial and repeat practitioner surveys is presented in Table 4.

**Table 5:** Comparison of initial and repeat clinician survey findings on D-dimer knowledge and practice Comparison of findings from the initial clinician survey (n=48) and repeat clinician survey (n=33) assessing knowledge and practice related to D-dimer testing in suspected pulmonary embolism. The table shows the proportion of respondents who correctly identified the local D-dimer cut-off (250 µg/L), correctly identified the local assay as D-dimer units (DDU), and reported regular use of age-adjusted D-dimer testing. Changes are expressed in percentage points (pp). Data are presented as N (%). Changes are expressed in percentage points (pp). DDU - D-dimer units

Measure	Initial survey, n=48	Repeat survey, n=33	Change
Correctly identified local D-dimer cut-off (250 µg/L)	33 (68.8%)	23 (69.7%)	+0.9 percentage points
Correctly identified local assay as DDU	2 (4.2%)	21 (63.6%)	+59.4 percentage points
Reported regular use of age-adjusted D-dimer	21 (43.8%)	18 (54.5%)	+10.7 percentage points

Factors Influencing Clinical Decision-Making

Fear of missing a diagnosis was reported as an important factor influencing CTPA requesting by 32/48 respondents (66.7%). Clinical impression was reported as taking precedence over formal scoring systems by 24/48 respondents (50.0%). Other reported triggers for imaging included a high Wells score in 14/48 responses (29.2%) and an elevated D-dimer result alone in 10/48 (20.8%).

The main reported challenges in CTPA decision-making were fear of missing a diagnosis in 32/48 responses (66.7%), uncertainty regarding Wells score application in 8/48 (16.7%), time pressure in 5/48 (10.4%), and other contextual factors in 3/48 (6.3%). Participants also identified the need for regular updates on diagnostic pathways, clearer guidance regarding senior review and more standardised protocols across departments.

Integration of Clinical Audit and Practitioner Survey Findings

Retrospective application of the age-adjusted D-dimer threshold increased specificity from 14.8% to 23.4%, while sensitivity decreased from 87.8% to 86.2%. False-positive classifications decreased from 813 to 731, representing 82 fewer false-positive classifications and a relative reduction of 10.1%. However, false-negative classifications increased from 23 to 26. Because this analysis was conducted in a CTPA-selected cohort and was not consistently restricted to patients with PE-unlikely or non-high pre-test probability, it should not be interpreted as validation of an age-adjusted D-dimer rule-out pathway.

The initial practitioner survey identified important barriers to implementation. Regular use of age-adjusted D-dimer thresholds was reported by 21/48 respondents (43.8%), while 6/48 (12.5%) were unfamiliar with the concept. In addition, 29/48 respondents (60.4%) were unsure whether the local laboratory used D-dimer units (DDU) or fibrinogen-equivalent units (FEU). Variation in knowledge of the local threshold and age-adjustment method was also reflected in errors in scenario-based calculations.

In the repeat cross-sectional survey, regular use of age-adjusted D-dimer thresholds was reported by 18/33 respondents (54.5%), unfamiliarity with the concept by 1/33 (3.0%), and correct identification of the locally used DDU assay by 21/33 (63.6%). Although assay knowledge and self-reported use were higher in the repeat survey, the initial and repeat surveys comprised independent, unpaired samples. Therefore, the observed differences cannot be attributed causally to the educational intervention.

Fear of missing a diagnosis was also frequently reported as influencing CTPA requesting, suggesting that clinical decision-making was not based solely on formal risk-stratification tools. This finding highlights the importance of clear local guidance, continued education and consistent application of validated diagnostic pathways.

For example, in a 75-year-old patient, the age-adjusted D-dimer threshold using a DDU assay would be 375 µg/L DDU (75 × 5). Variation in responses to the calculation-based question further demonstrated the importance of understanding the locally used assay and the corresponding age-adjustment formula.

## Discussion

This retrospective clinical audit and practitioner survey study provides insight into the classification performance of age-adjusted D-dimer thresholds and the practical challenges associated with their implementation in patients investigated for suspected pulmonary embolism (PE). In the diagnostic-analysis cohort, the age-adjusted threshold increased specificity from 14.8% to 23.4% and overall accuracy from 26.8% to 33.7%, while sensitivity decreased from 87.8% to 86.2%. False-positive classifications decreased from 813 to 731, representing 82 fewer false-positive classifications and a relative reduction of 10.1%. However, false-negative classifications increased from 23 to 26.

These results represent retrospective reclassification within a CTPA-selected cohort and should not be interpreted as demonstrating that imaging would have been safely avoided. The study did not assess a complete pre-test-probability-based diagnostic pathway, actual CTPA utilisation or subsequent thromboembolic outcomes among patients managed without imaging. Previous studies have suggested that appropriate use of age-adjusted D-dimer within validated diagnostic pathways may reduce unnecessary imaging, healthcare costs and radiation exposure [12-16]. However, the present findings do not directly demonstrate these outcomes.

The initial practitioner survey identified several potential barriers to clinical implementation. Twenty-nine of 48 respondents (60.4%) were unsure whether their laboratory used D-dimer units (DDU) or fibrinogen-equivalent units (FEU), potentially contributing to calculation errors and inconsistent application of age-adjusted thresholds. Knowledge of the locally used D-dimer threshold and the appropriate age-adjustment formula also varied. In the repeat cross-sectional survey, 21/33 respondents (63.6%) correctly identified DDU as the locally used assay, and regular use of age-adjusted D-dimer was reported by 18/33 (54.5%), compared with 21/48 (43.8%) in the initial survey. However, because the surveys comprised independent, anonymous and unpaired samples, these differences cannot be attributed causally to the educational intervention.

Clinical decision-making was also influenced by factors beyond formal risk-stratification tools. Fear of missing a diagnosis was reported as an important influence on CTPA requesting by 32/48 respondents (66.7%) [17-19]. Clinical impression was reported as taking precedence over formal scoring systems by 24/48 respondents (50.0%), demonstrating the continuing role of clinical judgement alongside validated tools such as the Wells score [20-22].

The physiological findings should be interpreted cautiously. PE positivity was higher among patients with an oxygen saturation of 90%-94% than among those with an oxygen saturation below 90%. Similarly, patients with a NEWS2 score of ≥10 had a lower observed PE positivity rate than those in the NEWS2 5-6 and 7-9 categories. These descriptive findings do not demonstrate independent associations, as severe physiological derangement may reflect a broad range of acute pathologies and no multivariable adjustment was performed.

The repeat survey demonstrated higher assay knowledge and self-reported use of age-adjusted D-dimer than the initial survey. Although causality cannot be established, the findings identify areas that may benefit from continued education, including assay-specific D-dimer interpretation, appropriate application of age-adjusted thresholds and adherence to pre-test-probability-based PE diagnostic pathways [23-25]. CTPA also identified alternative pathologies in a substantial proportion of PE-negative patients [26,27]. However, the exact alternative-diagnosis category totals require reconciliation with the source dataset before the overall number and percentage can be reported.

This study has several strengths. It included a relatively large real-world cohort of 1499 consecutive records and evaluated the locally used DDU assay. Reanalysis of the source data incorporated both numerical D-dimer results and valid censored results reported below the assay measurement threshold, enabling reconstruction of separate 2 × 2 contingency tables for the standard and age-adjusted thresholds. The inclusion of practitioner survey data also provided information about knowledge gaps and potential implementation barriers. Educational and quality-improvement interventions have previously been shown to support adherence to evidence-based PE diagnostic pathways [28,29], although the effectiveness of the intervention used in this study cannot be established from the unpaired surveys.

This study also has several important limitations. First, it was a single-centre retrospective clinical audit restricted to patients selected for CTPA, introducing selection and spectrum bias. Patients in whom PE was excluded clinically or through D-dimer testing without imaging were not included. Consequently, the findings cannot determine the number of CTPA examinations that would have been avoided through application of an age-adjusted threshold. Second, no three-month follow-up of patients managed without imaging was undertaken; therefore, the study cannot establish the safety of withholding imaging based on an age-adjusted D-dimer strategy.

Third, D-dimer results were unavailable or uninterpretable in 349/1499 records (23.3%). Because D-dimer testing was determined by routine clinical practice rather than by a study protocol, this missingness may not have been random and may reflect differences in pre-test probability or clinical decision-making. A further eight records without a definitive CTPA reference-standard outcome were excluded, leaving 1142 records in the diagnostic-performance analysis. Missing Wells score and physiological data also reduced the denominators for some analyses.

Fourth, the diagnostic analysis was not consistently restricted to patients with PE-unlikely or non-high pre-test probability because complete pre-test-probability data were unavailable. The findings therefore do not validate the age-adjusted threshold as a standalone PE rule-out strategy. Although specificity increased, sensitivity decreased from 87.8% to 86.2%, with three additional false-negative classifications.

Fifth, the initial and repeat practitioner surveys were relatively small, voluntary, anonymous and unpaired, involved different sample sizes, and did not include a control group. Response and selection bias are therefore possible, and observed differences between the survey samples cannot be attributed causally to the educational intervention. Finally, no post-intervention patient-level clinical re-audit was performed.

## Conclusions

Retrospective application of an age-adjusted D-dimer threshold in this CTPA-selected cohort increased specificity and reduced false-positive classifications, with a small reduction in sensitivity and three additional false-negative classifications. Because the analysis was not consistently restricted to patients with PE-unlikely or non-high pre-test probability, it does not validate a D-dimer-based PE rule-out pathway. Furthermore, actual CTPA utilisation and subsequent thromboembolic safety were not assessed.

In the repeat unpaired cross-sectional survey, assay knowledge and self-reported use of age-adjusted D-dimer were higher than in the initial survey; however, causality cannot be inferred. These exploratory findings support further prospective evaluation of an assay-specific, pre-test-probability-based diagnostic pathway with clinical follow-up.

## Appendices

Appendix 1: Practitioner Questionnaire

The following questionnaire was distributed to healthcare professionals involved in the assessment and management of patients with suspected pulmonary embolism (PE).

1. What is your current role?*

Junior Doctor

Senior House Officer

Registrar

Consultant

Other: __________

2. In which department do you primarily work?*

Emergency Department

Acute Medicine

General Medicine

Other: __________

3. What is the standard D-dimer cut-off for PE at your Trust?*

250 µg/L

500 µg/L

4. Do you know which D-dimer assay your hospital uses?*

Fibrinogen equivalent units (FEU)

D-dimer units (DDU)

Not sure

5. Do you use age-adjusted D-dimer cut-offs in your practice?*

Yes, regularly

Yes, sometimes

No

Not familiar with age-adjusted cut-offs

6. If you use age-adjusted D-dimer, how did you learn about it?*

Local guidelines

Senior colleagues

Medical school/training

Research papers

Other: __________

7. Age-adjusted D-dimer calculations apply to patients aged ≥ ____ years.*

45

50

55

60

8. Case 1*

75-year-old woman with a low Wells score.

Your calculated age-adjusted D-dimer cut-off (ng/mL):

9. Case 2*

A 68-year-old woman presents with acute-onset dyspnoea.

History: Breast cancer on chemotherapy Heart rate: 88 beats/min Blood pressure: 135/85 mmHg Oxygen saturation: 94% on room air Wells score: 3 points

What is the appropriate next step?

Order CTPA directly due to active cancer

Check D-dimer using an age-adjusted threshold

Check D-dimer using the standard threshold

Observe the patient due to stable vital signs

10. What is your biggest challenge when deciding about CTPA?*

Time pressure to make a decision

Uncertainty about Wells score

Patient preference/demands

Fear of missing the diagnosis

Other: __________

11. Which factor most often leads you to order CTPA?*

High Wells score

Elevated D-dimer

Patient anxiety

Overall clinical impression, independent of formal scoring

Other: __________

12. Which resources would help improve your PE assessment? Select all that apply.*

Quick-reference posters in assessment areas

Electronic ordering system with built-in decision support

Access to rapid senior review for complex cases

Regular updates on PE pathway changes

Structured teaching sessions on PE assessment

Clear guidance on when to discuss cases with senior clinicians

Regular audit of PE pathway compliance

Standardised documentation templates

Other: __________
